# p21 and CD166 as predictive markers of poor response and outcome after fluorouracil-based chemoradiotherapy for the patients with rectal cancer

**DOI:** 10.1186/1471-2407-14-241

**Published:** 2014-04-04

**Authors:** Sung Hoon Sim, Mi-Hyun Kang, Yu Jung Kim, Keun-Wook Lee, Duck-Woo Kim, Sung-Bum Kang, Keun-Yong Eom, Jae-Sung Kim, Hye Seung Lee, Jee Hyun Kim

**Affiliations:** 1 Department of Internal Medicine, Seoul National University Bundang Hospital, Seoul National University College of Medicine, 82, Gumi-ro 173 beon-gil, Bundang-gu, Seongnam 463-707, Korea; 2 Medical Science Research Institute, Seoul National University Bundang Hospital, Seoul National University College of Medicine, Seongnam, Korea; 3 Department of Surgery, Seoul National University Bundang Hospital, Seoul National University College of Medicine, Seongnam, Korea; 4 Department of Radiation Oncology, Seoul National University Bundang Hospital, Seoul National University College of Medicine, Seongnam, Korea; 5 Department of Pathology, Seoul National University Bundang Hospital, 82, Gumi-ro 173 beon-gil, Bundang-gu, Seongnam 463-707, Korea

**Keywords:** Rectal cancer, Chemoradiotherapy, Cancer stem cell, p21, CD166

## Abstract

**Background:**

Pre-operative chemoradiotherapy (CRT) is the standard treatment in clinical stage T3/4 or node positive rectal cancer. However, there are no established biomarkers that can predict the pathological response and clinical outcome to CRT.

**Methods:**

Immunohistochemical staining was performed in tissue arrays constructed from core tissue specimens taken before treatment and from operative specimens from 112 patients who received 5-FU based pre-operative CRT and surgery. Expression of Ki67, TS, BAX, EpCAM, p53, p21, EGFR, CD44, CD133, CD166, HIF1α and ALDH1 were assessed and correlated with tumor regression grades and disease free survival.

**Results:**

Of the 112 patients (M/F 74/38, median age: 62), 20 (17.9%) patients achieved pathologic complete remission (pCR). In analyzing the associations between marker expressions and tumor regression grades, high p21 expression at the pretreatment biopsy was significantly associated with non-pCR (p = 0.022) and poor disease free survival (median DFS - low vs high p21: 75.8 vs 58.1 months, p = 0.002). In the multivariate analysis, high p21 expression level at the pre-treatment biopsy was significantly associated with poor DFS (p = 0.001, HR 6.14; 95% CI 2.03, 18.55). High CD166 expression level at the pretreatment biopsy was also associated with poor DFS (p = 0.003; HR 5.61; 95% CI 1.81, 17.35).

**Conclusion:**

These show high p21 and CD166 expression at the pretreatment biopsy were associated with tumor regression and poor prognosis in patients treated with 5-FU based CRT. Larger, prospective and functional studies are warranted to determine the role of p21 and CD166 as predictive biomarker of response to CRT.

## Background

Since the report of CAO/ARO/AIO-94 trial showing an improved local recurrence rate and reduced toxicity with preoperative chemoradiotherapy (CRT), pre-operative CRT has become the standard treatment option for clinical stage T3/4 or node positive rectal cancer [1]. However, many patients still suffer recurrence and death after preoperative CRT and surgery, especially those who do not respond to the preoperative CRT. The patients who achieved complete regression after preoperative CRT attained a 5-year DFS of 86%, whereas patients who showed low grade of regression showed a 5-year DFS of not more than 63%, reappraising the need for a predictive marker of response to preoperative CRT [2].

There have been numerous reports on clinical and pathological biomarkers that can predict response to preoperative CRT, suggesting p53, p21, Ki67, bax, bcl-2, thymidylate synthase, etc. as predictive markers of response to preoperative CRT [3-7]. However, those studies suffer from a relatively small number of patients, their retrospective nature and limited availability of archived samples. There are still no validated biomarkers that can predict the response to CRT yet.

Recently, the cancer stem cell hypothesis has shed light on treatment resistance and recurrence of tumors, although the nature of these cells has not been identified clearly [8,9]. The cancer stem cell is thought to be dormant and resistant to conventional chemotherapy and radiation therapy, which can be attributed to treatment failure [10]. Several markers of cancer stem cells have been suggested in various types of cancers in which those markers may be associated with the response to chemotherapy and radiotherapy and disease free survival. However, few studies have addressed this issue of cancer stem cell markers as predictive markers for pathologic responses and treatment outcome in rectal cancer [4,11].

The purpose of our study was to identify predictive markers, in pre-treatment biopsies, of pathologic complete response (pCR) to preoperative CRT and disease free survival (DFS) after preoperative CRT and surgery.

## Methods

### Study design and statistical analysis

Using a prospectively maintained colorectal cancer database, patients who met the eligibility criteria were retrospectively enrolled in this study. Patients were eligible if 1) pathologically diagnosed with rectal adenocarcinoma at Seoul National University Bundang Hospital between Jun. 2003 and Dec. 2008, 2) the patients were consent with the use of pathology slides for research at the time of diagnosis 3) clinical stage T3/T4 and/or node positive by rectal MRI and/or endo-rectal ultrasonography, 4) received 5-FU-based CRT and surgical resection with curative aim and 5) had preoperative biopsy slides available.

All candidate variables for histologic marker analysis were p53, Ki67, TS, BAX, HIF1α, ALDH1, CD166, p21, EpCAM, CD44, CD133, which were selected due to potential candidates for cancer stem cell markers or histologic prognostic factors according to recent research on rectal cancer, with consideration of and technical availability [7,11-17].

The end point was to identify predictive markers, in pre-treatment biopsies, of pathologic complete response (pCR) to preoperative CRT and disease free survival (DFS) after preoperative CRT and surgery.

### Patients

The patients who were diagnosed with rectal cancer were retrospectively enrolled in this study. Eligible patients received pelvic radiotherapy with a dose of 45 Gy followed by a primary tumor boost of 5.4 Gy over a period of 5.5 weeks. Patients were given choices between capecitabine 825 mg/m^2^ twice daily throughout the radiation period or intravenous bolus 5-FU (400 mg/m^2^ daily injection for 4 days) and leucovorin (20 mg/m^2^ daily injection for 4 days), or to participate in a clinical trial. Surgery was performed approximately 6 weeks after completion of CRT. Postoperative chemotherapy was given to patients in stages 2 and 3 for an additional 4 months either with bolus 5-FU and leucovorin or 5-FU, leucovorin and oxaliplatin (FOLFOX-6). Patients were assessed every 6 months for follow-up, with a physical examination, measurements of carcinoembryonic antigen levels, and chest and abdominopelvic CT scans for 5 years after surgery.

### Specimen characteristics

Tissue samples before (pre-operative endoscopic biopsy) and after (surgical specimens) CRT were analyzed. The representative core tissue specimens (2 mm in diameter) were taken from individual paraffin blocks and rearranged in new tissue array blocks using a trephine apparatus (Superbiochips Laboratories, Seoul, Korea).

### Assay methods

#### Immunohistochemistry and evaluation

Array slides were immunohistochemically labeled with 11 commercially available antibodies: p53 (1:200, DAKO Glostrup, Denmark), Ki67 (1:100, DAKO Glostrup, Denmark), TS (1:50, Thermo Scientific, MA, USA), BAX (1:200, Epitomics, CA, USA), HIF1α (1:30, BD Biosciences, MA, USA), ALDH1 (1:1000, BD Biosciences, MA, USA), p21(1:300, Spring bioscience, CA, USA), EPCAM (1:200,abcam,MA,USA), CD44 (1:30 dilution; BD Biosciences, MA, USA), CD166 (1:100, Leica, Newcastle, UK) and CD133 (1:70, Abcam, ,MA,USA). Antigen retrieval was performed by immersing the slides in citrate buffer (pH 6.0) and microwaving them for 10 minutes. Nonreactive sites were blocked using 1% horse serum in Tris-buffered saline (pH 6.0) for 3 minutes. Primary antibodies were applied, and antibody binding was detected using the avidin-biotin peroxidase complex (Universal Elite ABC kit PK-6200; Vectastain, Burlingame, CA) and diaminobenzidine tetrahydrochloride solution (Kit HK 153-5 K; Biogenex, San Ramon, CA).

Immunohistochemical stains for p53, Ki67, TS, BAX, HIF1α, ALDH1, CD166, p21, EpCAM, CD44, and CD133 were performed in tissue arrays constructed from core tissue specimens taken before treatment and from operative specimens.

For p53, Ki67, TS, ALDH1, CD166, CD44, bcl2, EGFR, and CD133, normal colorectal epithelial cells were used as internal negative controls. We performed immune-staining without primary antibody for negative control. Location of staining; nucleus, membranous and/or cytoplasmic, were recorded in supplemental table (Additional file 1: Table S1). Only tumor cells were counted and cells stained on membrane were considered positive for CD 166 [11].

**Figure 1:**
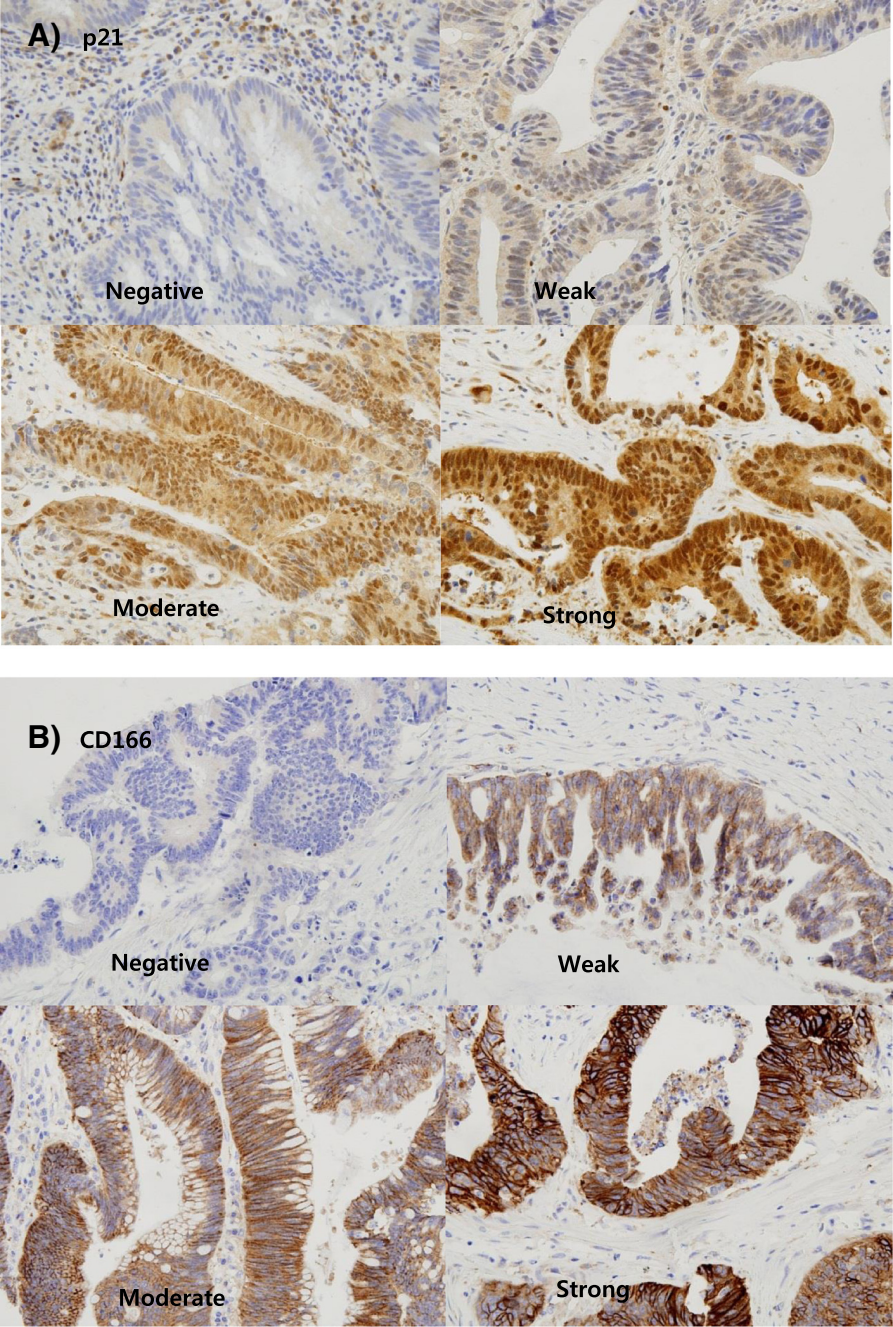
**Immunohistochemical staining of p21 and CD166 (x400). A)** Staining intensity of p21 **B)** Staining intensity of CD166.

The immune-reactivity was recorded as a total percentage (%) of positive cells for Ki67. For the other markers, the staining intensity was scored as follows: none, 0; weak, 1; moderate, 2; intense, 3 (Figure 1). If the staining intensity was heterogeneous in a section, it was scored based on that which was predominantly observed. The percentages of positive cells were assigned to one of five categories for protein expression: 0, 0-5%; 1, 5–25%; 2, 25–50%; 3, 50–75%; 4, 75-100%. The two scores were then multiplied to produce a weighted score for each tumor specimen [4]. Therapeutic responses to CRT were assessed with the Dworak Grades [18].

### Statistics

Frequency data were analyzed with the Chi-square test. To obtain the appropriate cut-off scores for each marker, receiver-operating characteristic (ROC) curve analysis was performed for pathologic complete response [19].The cut-off score was determined at the point of maximizing the sum of sensitivity and specificity. Survival analysis was done with the Kaplan–Meier method, and the groups were compared with the log-rank test. Univariate and multivariate analyses were performed based upon the logistic regression or Cox regression model with forward conditional variable entry (p < 0.05) and removal (p > 0.10). A two sided value of p < 0.05 was considered as statistically significant.

### Ethics

The study protocol was reviewed and approved by the Institutional Review Board of Seoul National University Bundang Hospital. The recommendations of the World Medical Association Declaration of Helsinki for Ethical Principles for Medical Research Involving Human Subjects were also followed.

## Results

### Patients’ characteristics

One hundred and sixty one consecutive patients who were diagnosed with rectal cancer and received 5-FU based pre-operative CRT were identified from the database. Among those who received preoperative CRT, 12 patients did not undergo surgery due to poor medical conditions or follow-up loss and 8 patients had initially metastatic or double primary malignant diseases. Eight patients were at stage I. For two patients, radiation therapy was terminated early due to complications and 19 patients did not have any initial pathologic slides available for this study. Finally, 112 patients were enrolled (Additional file 1: Figure S1). Their median age was 62 years (33 – 82). The characteristics of the patients are presented in Table 1. Chemotherapy given during the CRT phase was as follows: 49 received bolus 5-FU and leucovorin, 5 received 5-FU continuous infusion, 44 received capecitabine, 4 received capecitabine with irinotecan and cetuximab, and 10 patients received capecitabine with oxaliplatin. Low anterior resection (95.5%) or abdominoperineal resection (4.5%) was performed at a median of 6.7 weeks (range; 5.1 to 9 weeks) after CRT. Out of 112 patients, 20 (17.9%) patients achieved pathologic complete remission (pCR).

**Table 1:** Patient demographics and clinical characteristics

**Characteristics**	**Characteristic**	**No (%)**
**Sex**	Male	74 (66.1%)
	Female	38 (33.9%)
**Age**	Range, median	33 ~ 82, 62
**ECOG Performance status**	0	3 (2.7)
	1	103 (92.0)
	2	5 (4.5)
	3	1 (0.9)
**Histology**	Adenocarcinoma	111 (99.1%)
	AdenoSquamous carcinoma	1 (0.9%)
**Differentiation**	Well differentiated	20 (17.9)
	Moderatly differentiated	86 (76.8)
	Poorly differentiated	6 (5.4)
**cT**	1	0 (0%)
	2	1 (1%)
	3	97 (86.5%)
	4	14 (12.5%)
**cN**	0	46 (41.1%)
	1	48 (42.9%)
	2	18 (16.1%)
**Turmor regression grade (Dworak)**	1	18 (16.1%)
	2	48 (42.9%)
	3	26 (23.2%)
	4	20 (17.9%)
**ypT**	0 (total regression)	20 (17.9%)
	1	3 (2.7%)
	2	31 (27.7%)
	3	55 (49.1%)
	4	1 (0.9%)
	Unknown	2 (1.7%)
**ypN**	0	79 (70.5%)
	1	23 (20.5%)
	2	10 (8.9%)
	Unkonwn	2 (1.7%)
**Circumferential resection margin**	Median, range (cm)	1.0 (0.01,2.5)

### Score distributions

All the pre-operative biopsy samples were available but post–operative surgical samples were available in only 75 patients due to the operations taking place in other hospitals, poor specimen quality or total tumor regression after CRT. All marker expression scores were classified into high or low using cut-off values defined as described (Additional file 1: Table S2). The sensitivity of each marker expression according to the cut-off value ranged from 0.099 to 0.637 but the specificity ranged from 0.5 to 1.0.

The mean immune reactivity scores of p53, Ki67, HIF1α, CD44 and CD133 were higher in the pre-operative biopsy samples than in the post-operative surgical samples (Additional file 1: Table S3).

### Association between marker expression level and complete pathologic response

The pathologic complete response was significantly associated with Ki67 and p21 expression (p = 0.022. and 0.022, respectively). Among the clinical factors, clinical stage III and high CEA level exhibited significantly poor complete pathologic response (p = 0.001 and 0.011, respectively, Additional file 1: Table S4). In the multivariable analysis, clinical stage III, high CEA level and high p21 expression were independently associated with poor pathologic responses (Odd ratio, 0.199, 0.422, 0.127 in clinical stage, CEA level and p21) (Table 2).

**Table 2:** Multivariable analysis of marker expression and clinical factors with pathologic response

**Variables**	**p-value**	**OR (95% CI)**
Clinical stage	0.028	0.199 (0.056,0.708)
Pre-operative CEA levels	0.044	0.422 (0.084,2.120)
Ki67	0.999	NA
p21	0.021	0.127 (0.022,0.729)

NA: not applicable :low Ki67 – no pCR observed.

### Association between marker expression levels and disease free survivals

**Figure 2:**
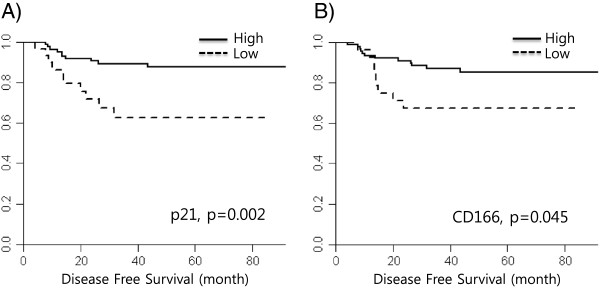
**Disease free survival according to expression of A) p21 and B) CD166.** The DFS of high p21 group was 58.9 months (95% CI 44.4, 71.7), low p21, 75.8 months (95% CI 70.8, 80.8), high CD166, 61.6 month (95% CI 49.5, 73.8) and low CD166, 75.2 months (95% CI 69.6, 80.8).

The median disease free survival was not reached after a median follow-up of 48.1 months. Disease recurrence was observed in 21 patients (18.8%). In the analysis between clinical factors and disease free survival, low tumor regression grade, high pathologic T and high pathologic N stage (ypT and ypN) were significantly associated with short disease free survival (p < 0.001). In the marker analysis, high expressions of CD166 and p21 in the pretreatment biopsy specimens were associated with short disease free survival (p = 0.045 and 0.002. respectively). The Ki67 expression, which showed a significant association with the pathologic complete response, was not associated with disease free survival (p = 0.667, Additional file 1: Table S5). In the multivariable analysis, tumor regression grade, ypN, CD166 and p21 expression levels in pre-operative biopsy samples were significantly associated with disease free survival, showing that a high expression level of CD166 and p21 exhibited shorter disease free survival (HR 5.614, p = 0.003 in CD166, HR 6.146, p = 0.001 in p21, Table 3, Figure 2). There was no significant correlation between the p21 expression levels in the pre-operative samples and clinical characteristics including age, sex, histologic differentiation and clinical stage (Additional file 1: Table S6).

**Table 3:** Multivariable analysis of cell markers and disease free survival (DFS)

**Variables**	**HR (95% CI)**	**p-value**
Tumor regression grade 1	1	0.046
Tumor regression grade 2	0.257 (0.079,0.853)	0.024
Tumor regression grade 3	0.065 (0.007,0.604)	0.016
Tumor regression grade 4*	<0.001 (NA).	0.999
ypN0	1	0.001
ypN1	9.025 (2.276,35.792)	0.002
ypN2	15.542 (3.634,66.462)	0.001
CD166	5.614 (1.816,17.356)	0.003
p21	6.146 (2.035,18.559)	0.001

*No event (disease recurrence) was observed.

### Score analysis within the paired samples

In the analysis of scores within the paired samples, there was a significant score change in Ki67, HIF1α, ALDH1, CD166, EpCAM and CD44 but not in p53, TS, BAX, p21 and CD133. The expression scores of Ki67, HIF1α and CD44 decreased and those of ALDH1, CD166 and EpCAM increased in the post-operative specimens compared with the scores in the pre-operative specimens (Table 4, Additional file 1: Figure S2 and Figure S3,). The change in marker expression levels between before and after treatment showed no statistical significance on disease free survival (Additional file 1: Figure S4). Trends for longer survival with a decrease in CD166 and an increase in p21 levels were observed but the significance was marginal (p = 0.071 and 0.080 in CD166 and p21).

**Table 4:** Serial analysis of marker expression in patients who had both pre- and post-operative specimens (by Wilcoxon signed rank test)

**Markers**	**Mean score**		**P-value**
	**Pre-operative specimen**	**Post-operative specimen**	
p53	7.098	6.845	0.135
Ki67	48.098	21.555	0.001
TS	1.126	1.291	0.661
BAX	4.357	4.943	0.326
HIF1α	1.563	0.591	0.001
ALDH1	1.197	3.507	0.001
CD166	0.478	2.305	0.001
p21	6.357	7.281	0.090
EpCAM	9.985	12.0	0.001
CD44	1.394	0.859	0.028
CD133	3.943	4.309	0.193

## Discussion

The present study showed that high p21 expression level in the pre-CRT specimen was associated with poor pathologic regression and worse DFS after CRT. Our study also showed that expression levels of potential cancer stem cell markers, especially CD 166, increased after preoperative CRT and high expression of CD166 was significantly associated with poor DFS.

p21 is known as one of the cell cycle inhibitors which plays a role through the p53 dependent or independent pathway. However, little is known about its clinical significance, especially with regard to rectal cancer. In leukemogenesis, p21 expression in leukemic stem cell preserves the cell proliferation ability and protects against DNA damage by cell cycle inhibition [20]. Its dormancy with p21 expression may be associated with chemotherapy resistance. In addition, the role of p21 has been reported to be important in chemotherapy resistance in cell line experiments in colorectal cancer [21]. These reports support our results that the initial high expression of p21 was associated with poor clinical outcome. The trend for better disease free survival with the increase in p21 expression after chemoradiotherapy could be associated with increased tumor cell cycle inhibition.

In several clinical studies, the role of p21 expression as a prognostic or predictive factor on survival in rectal cancer is controversial. One study showed that the post-CRT p21expression level was associated with survival but not with the pre-CRT level [13]; however, the treatment was different from the recent standard pre-operative CRT. Other studies with recent pre-operative CRT reported that the pre-CRT p21 expression level had prognostic impact on survival but not the post-CRT level [22] which supports the results of our study that shows the consistent significance of the pre-CRT p21 level on predicting the pathologic response and survival outcome (p = 0.022 and p = 0.021 respectively).

We wanted to test the hypothesis that cancer cells expressing potential stem cell markers would be a poor predictive marker of response to preoperative CRT, and found that high expression of CD166, but not CD 44 or 133, was associated with poor survival outcome. The increased expression of CD 166 in the postoperative specimen after CRT was also notable. The proliferation index was higher in the pre-CRT specimens than in the postoperative specimens. However, expression of ALDH1,CD166 and EpCAM, which could represent the dormant stem cell portion, increased in the post-operative specimens which are in agreement with previous reports [23-25]. Although robust marker changes were not observed between pre- and post- tumors, the present study showed the trend for enrichment of cells expressing potential stem cell markers.

There have been numerous reports on the increase in cancer stem cell markers in post-chemotherapy and/or radiotherapy specimens in other organs such as breast cancer, linking the presence of cancer stem cells to resistance to chemotherapy. In colon cancer, CD133 was the first markers associated with cancer stem cell in mouse model [26]. Most reports used CD 133 or 44 as cancer stem cell markers [11,12,15,16]. However, other reports suggested that CD44, CD166 could be more robust identifiers than CD133 [16,27]. In rectal cancer, reports on the meaning of putative cancer stem cell markers in the chemotherapy and/or radiotherapy have been scarce.

CD166 is a kind of cell adhesion molecule and is widely expressed in various tissues, such as neurons, fibroblasts, endothelial cells, and keratinocytes [28]. Not much of its function is known in cancer. Several reports have shown that CD166 was involved in the tumor cell invasion process by activating the metalloproteinase cascade in response to extensive cell-to-cell and cell-to-matrix contacts and also suggested CD166 as a potential marker for tumor invasion and metastasis [28,29]. Other clinical reports also have suggested CD166 was a putative marker for radiation resistance and a poor prognosis, which corresponded with our results [11,30].

Other markers except p21 and CD166 did not show clinical significance in this study. However, many studies reported the clinical meaning of markers such as BAX TS CD133 CD44, EpCAM, ALDH1 or HIF1α [4,11,24,31]. This may be attributed to the different type of cancer and the lack of a standard method for evaluating those markers as well.

This study has several limitations. First, this was a retrospective study and the results were not validated in other patient groups. Second, all markers were evaluated based on protein expression levels, not on genetic levels, which means the results do not reflect molecular changes in cancer cells on a genetic level. Third, the method used for investigating these markers was IHC staining alone This method may show a wide variety due to methodological differences and needs standardization before our results can be widely applied. However, our study comprehensively evaluated marker expression using immunohistochemistry in an attempt to find predictive biomarkers of response to CRT in a relatively larger number of patients than before, and in uniformly treated patients at a single center. The trend for enrichment of cells expressing potential stem cell markers after CRT is notable, which need further investigation for the possibility of treatment targeting these cells. Immunohistochemical staining is a widely used technique which means this result could have practical clinical applications. Although it may not be possible to characterize cancer stem cells by a single marker, our study results suggest that CD 166 and p21 can be used to select patients who would less likely benefit from preoperative CRT in rectal cancer. This study cannot overcome the defects that previous studies have. However, the results can call attention to the old but potential markers, p21 and CD166 as useful selection markers with new perspectives.

## Conclusion

In summary, high p21 expression was associated with non-complete pathologic response and poor disease free survival outcome in patients with 5-FU based CRT. High CD166 expression was associated with poor prognosis but the association with non-complete pathologic response was not significant. Larger, prospective trials and functional studies are warranted to determine the role of p21 as a predictive biomarker of response to chemoradiotherapy.

## Abbreviations

TS: Thymidylate synthase; BAX: bcl-2 associated X protein; EpCAM: Epithelial cell adhesion molecule; HIF1α: Hypoxia-inducible factor 1-alpha; ALDH1: Acetaldehyde dehydrogenase 1.

## Competing interests

The authors declare that they have no competing interests.

## Authors’ contributions

SHS: designed the concept of this study, performed the statistical analysis and drafted the manuscript. MHK: collected data and performed the statistical analysis. YJK, KWL, DWK, SBK, KYE, JSK: critically revised the manuscript. HSL, JHK: participated in its design and coordination, critically revised the manuscript. All authors read and approved the final manuscript.

## Pre-publication history

The pre-publication history for this paper can be accessed here:

http://www.biomedcentral.com/1471-2407/14/241/prepub

## Supplementary Material

Additional file 1: Table S1 Summary of immunohistochemical staining pattern. **Table S2.** Summary of AUC and ROC cut-off values. **Table S3.** Score distribution of all markers. **Table S4.** Univariable analysis of marker expressions and clinical factors with pathologic responses. (A) Association between marker expression level and complete pathologic responses (B) Association between clinical factors and complete pathologic responses. **Table S5.** Univariable analysis - cell markers and disease free survival (DFS). (A) Association between clinical factors and DFS. (B) Association between marker expression levels and DFS. **Table S6.** Association between p21 expressions in pre-operative samples and clinical characteristics. **Figure S1.** Flow diagram of patients included in the analysis. **Figure S2.** Serial changes of marker expression between pre- and post- chemoradiation treatment. **Figure S3.** Representative examples of immunohistochemical analyses of CD166 in pre-CCRT and post-operative specimens. These show increased CD166 expression levels after chemoradiation. **Figure S4.** Impact of marker expression change on disease free survival; solid line: longitudinal score increase, dashed line: longitudinal score decrease. Click here for file
