# Supplementary material for: p21 and CD166 as predictive markers of poor response and outcome after fluorouracil-based chemoradiotherapy for the patients with rectal cancer
Source: BMC Cancer. 2014 Apr 4;14:241. doi: 10.1186/1471-2407-14-241 (PMC4101833; doi:10.1186/1471-2407-14-241)
Supplement: Additional file 1: Table S1 — Summary of immunohistochemical staining pattern. Table S2. Summary of AUC and ROC cut-off values. Table S3. Score distribution of all markers. Table S4. Univariable analysis of marker expressions and clinical factors with pathologic responses. (A) Association between marker expression level and complete pathologic responses (B) Association between clinical factors and complete pathologic responses. Table S5. Univariable analysis - cell markers and disease free survival (DFS). (A) Association between clinical factors and DFS. (B) Association between marker expression levels and DFS. Table S6. Association between p21 expressions in pre-operative samples and clinical characteristics. Figure S1. Flow diagram of patients included in the analysis. Figure S2. Serial changes of marker expression between pre- and post- chemoradiation treatment. Figure S3. Representative examples of immunohistochemical analyses of CD166 in pre-CCRT and post-operative specimens. These show increased CD166 expression levels after chemoradiation. Figure S4. Impact of marker expression change on disease free survival; solid line: longitudinal score increase, dashed line: longitudinal score decrease. [file 1471-2407-14-241-S1.docx]

Supplement Tables and Figures

Table 1 Summary of immunohistochemical staining pattern

| marker | tumor |
| --- | --- |
| p53 | nucleus |
| Ki67 | nucleus |
| TS | cytoplasm ± nucleus |
| Bax | cytoplasm |
| HIF1alpha | nucleus |
| ALDH1 | cytoplasm ± nucleus |
| CD166 | membrane ± cytoplasm |
| p21 | nucleus |
| EpCAM | membrane ± cytoplasm |
| CD44 | membrane ± cytoplasm |
| bcl2 | nucleus |
| EGFR | membrane ± cytoplasm |
| CD133 | membrane ± cytoplasm |

Table 2. Summary of AUC and ROC cut-off values

| marker | AUC (95% CI) | Cut-off | Sensitivity/Specificity |
| --- | --- | --- | --- |
| p53 | 0.519(0.363,0.634) | 7.5 | 0.417,0.652 |
| Ki67 | 0.569(0.437,0.717) | 12.5 | 0.209,1.000 |
| TS | 0.503(0.374,0.621) | 2.5 | 0.121,0.950 |
| BAX | 0.525(0.349,0.643) | 7 | 0.300,0.756 |
| HIF1 | 0.559(0.408,0.701 | 0.5 | 0.637,0.500 |
| ALDH1 | 0.511(0.376,0.579) | 7 | 0.099,0.950 |
| CD166 | 0.560(0.455,0.639) | 0.5 | 0.257,0.850 |
| p21 | 0.615(0.516,0.736) | 8.5 | 0.311,0.950 |
| EpCAM | 0.589(0.462,0.660) | 8.5 | 0.275,0.900 |
| CD44 | 0.603(0.434,0.693) | 0.5 | 0.330,0.800 |
| CD133 | 0.537(0.434,0.660) | 1.5 | 0.319,0.900 |

Table 3. Score distribution of all markers

| Markers | Score | | | |
| --- | --- | --- | --- | --- |
|  | Pre-operative | | Post-operative | |
|  | mean | median | mean | median |
| p53 | 7.324 | 12.0 | 6.845 | 3.0 |
| Ki67 | 48.333 | 50.0 | 21.555 | 10.0 |
| TS | 1.108 | 0.0 | 1.291 | 0.0 |
| BAX | 4.081 | 4.0 | 4.943 | 4.0 |
| HIF1a | 1.495 | 1.0 | 0.591 | 0.0 |
| ALDH1 | 1.432 | 0.0 | 3.507 | 2.0 |
| CD166 | 0.468 | 0.0 | 2.305 | 1.0 |
| p21 | 6.271 | 6.0 | 7.281 | 8.0 |
| EpCAM | 10.180 | 12.0 | 12.0 | 12.0 |
| CD44 | 1.720 | 1.0 | 0.859 | 0.0 |
| CD133 | 4.090 | 3.0 | 4.309 | 4.0 |

Table 4. Univariable analysis of marker expressions and clinical factors with pathologic responses. (A) Association between marker expression level and complete pathologic responses (B) Association between clinical factors and complete pathologic responses

(A)

| Markers | Expression | non-pCR | pCR | p-value |
| --- | --- | --- | --- | --- |
| p53 | Low | 38 | 7 | 0.577 |
|  | High | 53 | 13 |  |
| Ki67 | Low | 19 | 0 | 0.022 |
|  | High | 72 | 20 |  |
| TS | Low | 80 | 19 | 0.690 |
|  | High | 11 | 1 |  |
| BAX | Low | 68 | 14 | 0.606 |
|  | High | 22 | 6 |  |
| HIF1a | Low | 33 | 10 | 0.254 |
|  | High | 58 | 10 |  |
| ALDH1 | Low | 82 | 19 | 0.687 |
|  | High | 9 | 1 |  |
| CD166 | Low | 66 | 17 | 0.393 |
|  | High | 25 | 3 |  |
| p21 | Low | 62 | 19 | 0.022 |
|  | High | 28 | 1 |  |
| EpCAM | Low | 25 | 2 | 0.149 |
|  | High | 66 | 18 |  |
| CD44 | Low | 30 | 4 | 0.297 |
|  | High | 61 | 16 |  |
| CD133 | Low | 29 | 2 | 0.056 |
|  | High | 62 | 18 |  |

(B)

| Clinical factors | Non- pCR | pCR | p-value |
| --- | --- | --- | --- |
| Age  ≤60  >60 | 43  59 | 9  11 | 0.888 |
| Sex  M  F | 63  29 | 11  9 | 0.249 |
| Histologic differentiation  Well or Moderate differentiation  Other differentiation | 88  4 | 18  2 | 0.309 |
| Clinical stage  II  III | 31  61 | 15  5 | 0.001 |
| CEA level  ≤2  >2 | 31  59 | 12  6 | 0.011 |

Table 5. Univariable analysis - cell markers and disease free survival (DFS). (A) Association between clinical factors and DFS. (B) Association between marker expression levels and DFS

|  | **No.** | | **Mean DFS**  **(95% CI)** | **p-value** |
| --- | --- | --- | --- | --- |
| Age  ≤60  >60 | 52  60 | 67.80 (59.64, 75.97)  74.80 (68.08, 81.52) | | 0.312 |
| Sex  M  F | 74  38 | 71.94 (65.77, 78.11)  70.23 (60.56, 79.90) | | 0.593 |
| Histologic differentiation  Well or Moderate differentiation  Other differentiation  Clinical stage  II  III | 106  6  46  66 | 71.95 (66.43,77.49)  75.47 (59.87,91.08)  73.56 (66.18, 80.95)  70.18 (62.82, 77.53) | | 0.769  0.400 |
| Tumor regression grade  1  2  3  4 | 18  48  26  20 | 38.44(27.06,49.81)  65.67(57.71,73.63)  83.52(78.78,88.27)  NA | | <0.001 |
| ypT  0(total regression)  1  2  3  4  ypN  0  1  2 | 20  3  31  55  1  79  23  10 | NA  NA  81.57 (75.62,87.53)  54.84 (47.23,62.45)  13.90 (13.90,13.90)  80.38 (76.68,84.79)  56.58 (45.44,69.63)  34.19 (16.73,51.65) | | <0.001  <0.001 |
| CEA level  ≤2  >2 | 43  65 | 75.92 (68.40,82.94)  69.41 (62.03,76.79) | | 0.164 |

(A)

(B)

|  | **No.** | **Mean DFS**  **(95% CI)** | **p-value** |
| --- | --- | --- | --- |
| p53  Low  High | 45  66 | 71.64 (63.63,79.65)  71.43 (64.33, 78.52 | 0.778 |
| Ki67  Low  High | 19  92 | 72.56 (60.46, 84.64)  71.49 (65.49, 77.48) | 0.667 |
| TS  Low  High  BAX  Low  High | 99  12  82  28 | 72.84 (67.33,78.34)  61.01 (42.33,79.68)  73.57 (67.64, 79.50)  68.08 (56.51, 79.56) | 0.363  0.524 |
| HIF1a  Low  High | 43  68 | 77.77 (70.97,84.57)  68.15 (60.91,75.40) | 0.133 |
| ALDH1  Low  High  CD166  Low  High  p21  Low  High  EpCAM  Low  High  CD44  Low  HIgh  CD133  Low  High | 101  10    83  28  81  29  27  84  34  77  31  80 | 72.85 (67.35,78.35)  62.52 (47.38,77.65)    75.20 (69.56,80.85)  61.66 (49.53,73.80)  75.81 (70.79,80.83)  58.09 (44.45,71.73)  65.18 (53.01,77.34)  74.68 (69.02,80.34)  67.28 (56.98,77.57)  73.56 (67.42,79.71)  64.14 (53.15,75.13)  74.65 (68.74,80.57) | 0.872  0.045  0.002  0.181  0.414  0.112 |

Table 6. Association between p21 expressions in pre-operative samples and clinical characteristics

| Clinical factors | | p21 | | p-value |
| --- | --- | --- | --- | --- |
|  |  | Low (n=81) | High (n=29) |  |
| Sex | M  F | 53  28 | 19  10 | 0.993 |
| Age (mean±SD) | | 60.6±10.3 | 59.4±12.6 | 0.603 |
| Histologic Differentiation | Well  Moderate  Poorly | 16  62  3 | 4  22  3 | 0.342 |
| cT | 1  2  3  4 | 0  1  72  8 | 0  1  23  6 | 0.280 |
| cN | 0  1  2 | 34  38  9 | 12  10  7 | 0.197 |
| Tumor regression Grade | 1  2  3  4 | 10  35  17  19 | 7  12  9  1 | 0.056 |
| ypT | 0  1  2  3  4 | 19  3  20  38  0 | 1  0  11  15  1 | 0.039 |
| ypN | 0  1  22 | 58  16  7 | 20  6  3 | 0.952 |

**Legends of Figures**

Figure 1. Flow diagram of patients included in the analysis.

Figure 2. Serial changes of marker expression between pre- and post- chemoradiation treatment.

Figure 3 Representative examples of immunohistochemical analyses of CD166 in pre-CCRT and post-operative specimens. These show increased CD166 expression levels after chemoradiation

Figure 4. Impact of marker expression change on disease free survival; solid line : longitudinal score increase , dashed line : longitudinal score decrease.

**Figure 1**

161 Patients

12 Patients: no surgery and/or follow up loss

8 Patients : initially metastatic and/or double primary malignant disease

2 Patients : early termination of radiation due to complication

19 Patients : no pathologic slides available

112 Patients

8 Patients: initial stage I

**Figure 2**


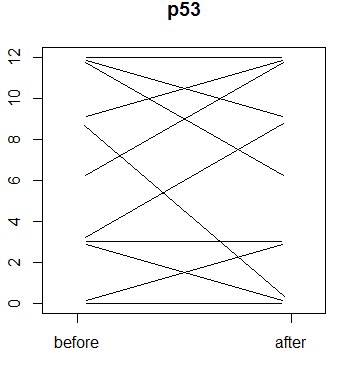

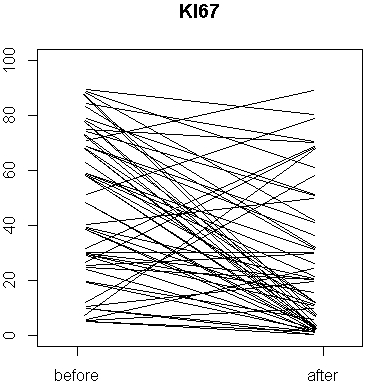

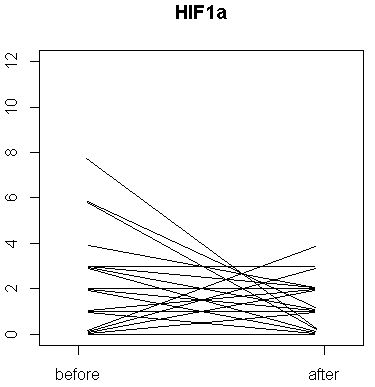

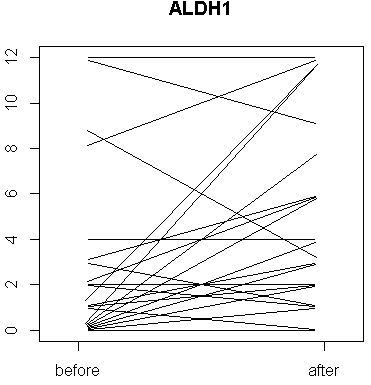

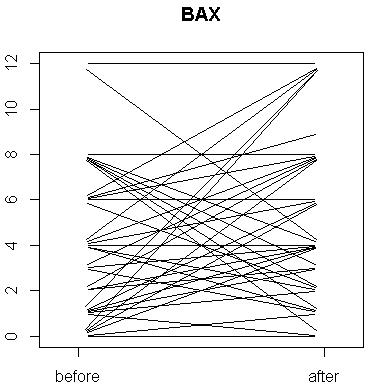


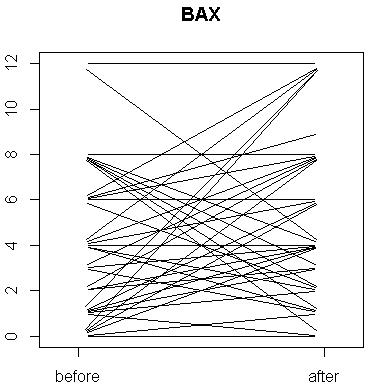


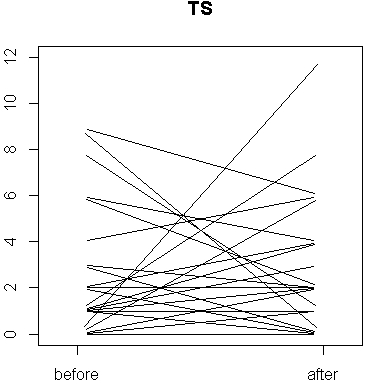


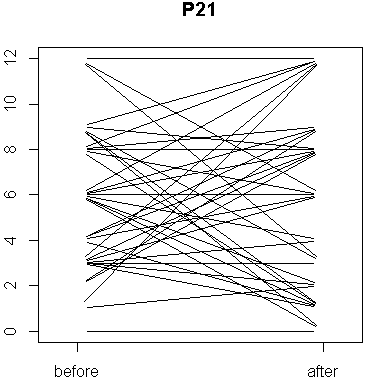

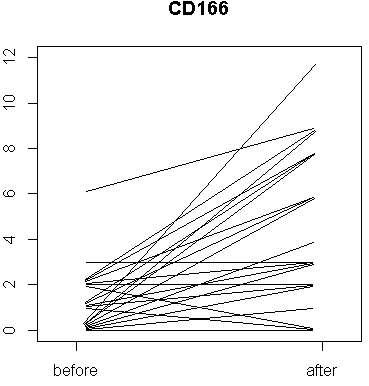


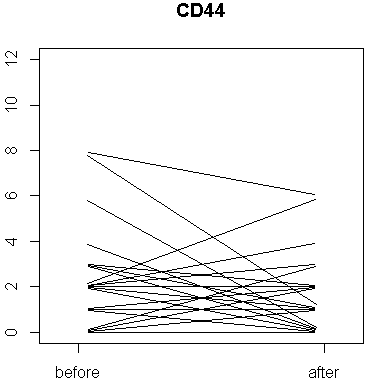

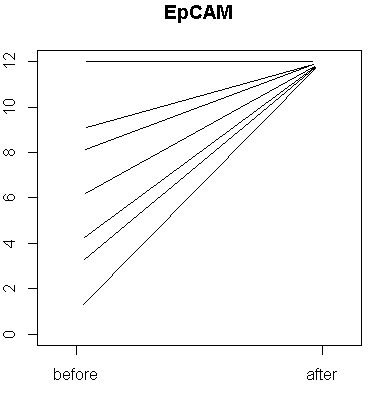


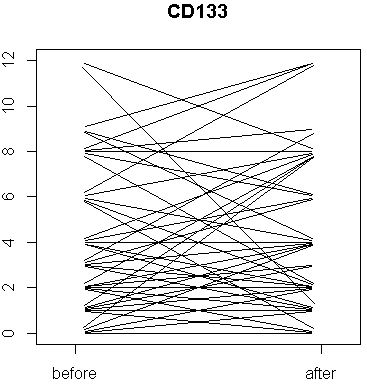


**Figure 3**


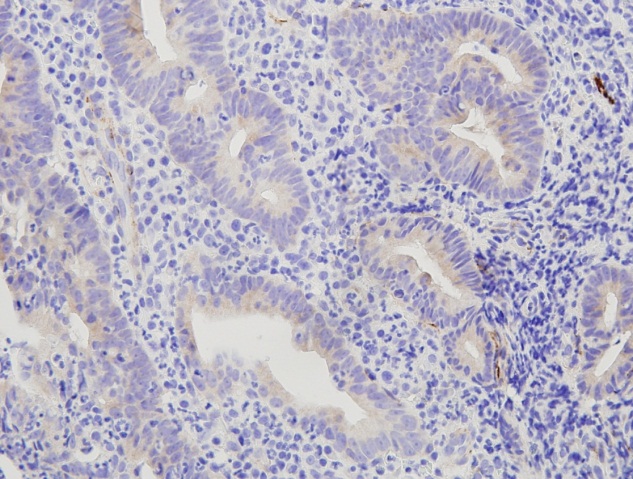

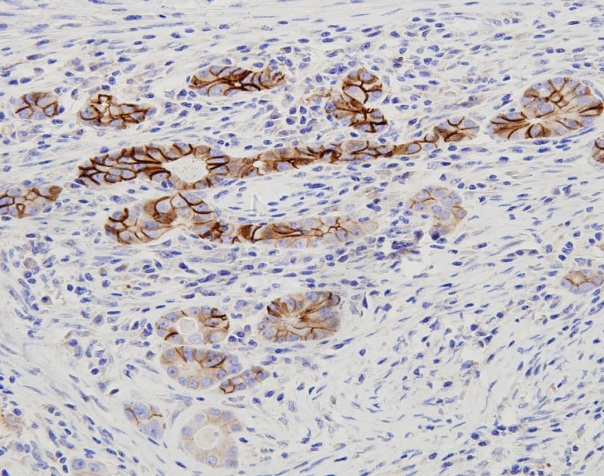


**Before**

**After**

**Figure 4**


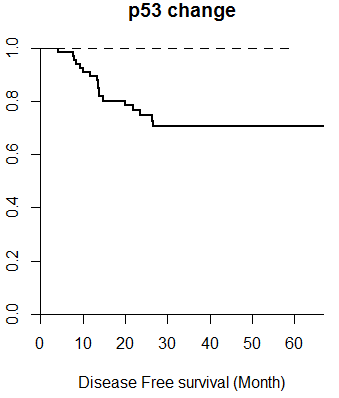

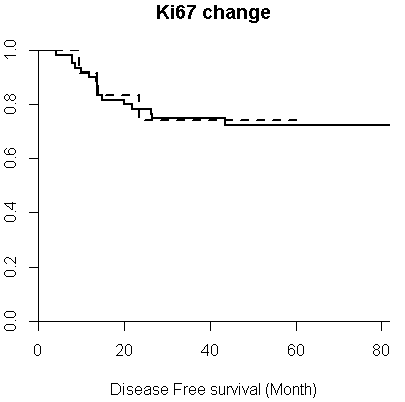


P=0.227

P=0.897


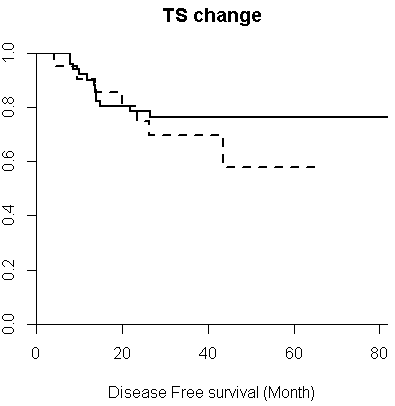

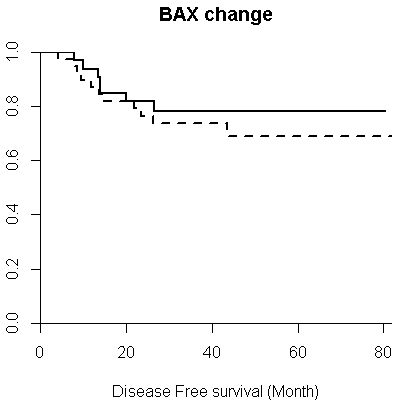


P=0.464

P=0.419


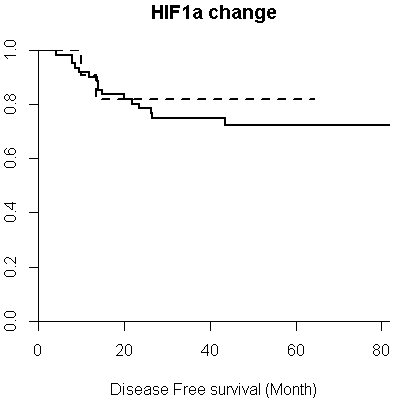

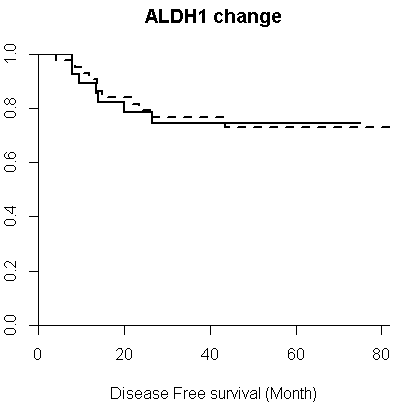


P=0.561

P=0.874


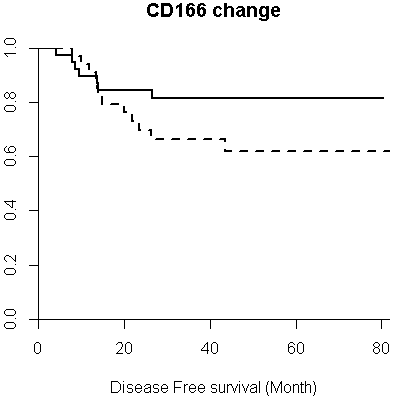

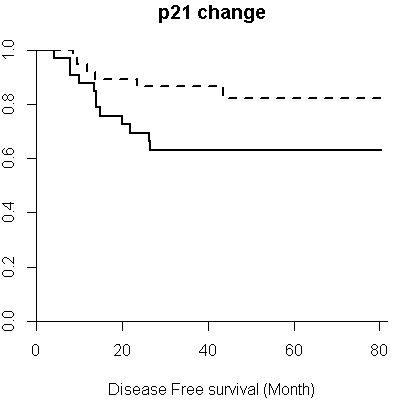


P=0.071

P=0.080


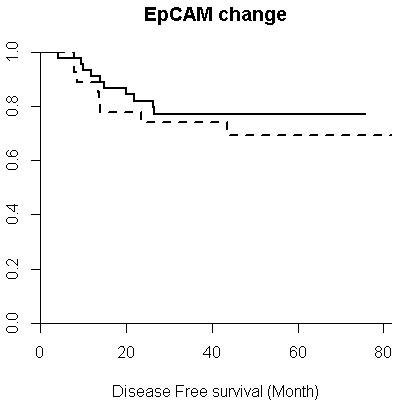


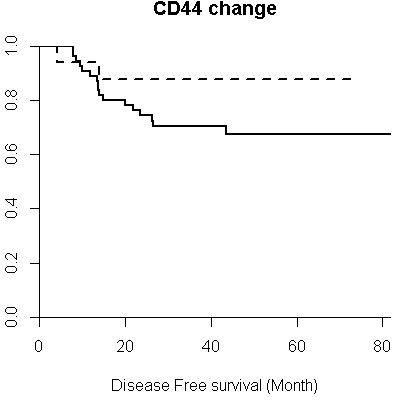


P=0.184

P=0.414


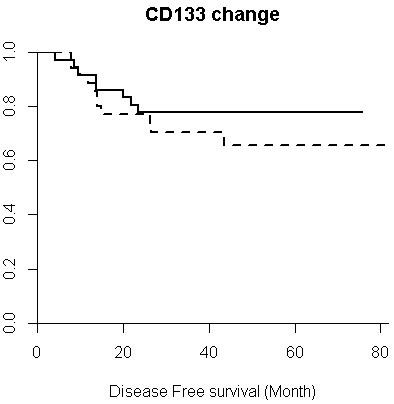


P=0.325
